# LncRNA DGCR5 plays a tumor-suppressive role in glioma via the miR-21/Smad7 and miR-23a/PTEN axes

**DOI:** 10.18632/aging.103800

**Published:** 2020-10-21

**Authors:** Zongze He, Juan Long, Chen Yang, Bo Gong, Meixiong Cheng, Qi Wang, Jian Tang

**Affiliations:** 1 Department of Neurosurgery, Sichuan Academy of Medical Sciences and Sichuan Provincial People’s Hospital, School of Medicine, University of Electronic Science and Technology of China, Chengdu 610072, Sichuan, China; 2 Institute of Chengdu Biology, Sichuan Translational Medicine Hospital, Chinese Academy of Sciences, Chengdu 610072, Sichuan, China; 3 Department of Laboratory Medicine, Sichuan Academy of Medical Sciences and Sichuan Provincial People’s Hospital, School of Medicine, University of Electronic Science and Technology of China, Chengdu 610072, Sichuan, China

**Keywords:** glioma, lncRNA DGCR5, miR-21/Smad7 axis, miR-23a/PTEN axis

## Abstract

Glioma is one of the most commonly diagnosed brain malignancies with a high cancer-related death rate in humans. The prognosis of glioma patients is still unsatisfactory. In the present study, we attempted to identify lncRNAs and miRNAs that might be related to NF-κB-mediated epithelial-mesenchymal transition in glioma cells based on online microarray expression profiles, and investigate the specific effects of lncRNA-miRNA-mRNA axes on glioma cell phenotypes. Herein, we identified lncRNA DGCR5 as a downregulated lncRNA in glioma that was negatively regulated by NF-κB1 in an NF-κB1 RE-dependent manner. LncRNA DGCR5 overexpression significantly inhibited the capacity of glioma cells to proliferate, migrate, and invade, whereas promoted the apoptosis of glioma cells. Moreover, lncRNA DGCR5 overexpression upregulated the epithelial marker E-cadherin while downregulating the mesenchymal marker VIM, as well as Snai2 and TWIST. Regarding the underlying molecular mechanisms, lncRNA DGCR5 could inhibit miR-21 and miR-23a expression, and miR-21 or miR-23a overexpression significantly reversed the tumor-suppressive effects of lncRNA DGCR5 overexpression. LncRNA DGCR5 exerted its tumor-suppressive effects through the DGCR5/miR-21/Smad7 and DGCR5/miR-23a/PTEN axes. In conclusion, lncRNA DGCR5 suppresses the capacity of glioma cells to migrate and invade via miR-21/Smad7, whereas it inhibits the proliferation and enhances the apoptosis of glioma cells through miR-23a/PTEN.

## INTRODUCTION

Glioma is one of the most commonly diagnosed brain malignancies and lethal cancers in humans [1]. Despite developments in glioma therapies, the prognosis of glioma patients is unsatisfactory [2, 3]. Therefore, investigating the underlying molecular mechanisms involved in glioma onset and progression will be essential for finding potential therapeutic targets.

Inflammation is a natural immune response to multiple stimuli, including infections, tissue injuries, or organ malfunction. Upon the persistent existence of the stimuli initiating the inflammatory responses, acute inflammation might progress to chronic one. Unlike the acute inflammation, which might be beneficial, chronic inflammation could cause damage to tissues, sometimes leading to cancer growth and contributing to tumorigenesis [4–7]. During inflammation, increased cytokines can trigger signaling cascades that activate NF-κB, which in turn, activates proliferation-related genes and anti-apoptotic genes [8]. In general, NF-κB is located at the junction of upstream inducers and downstream mediators of the EMT (epithelial-mesenchymal transition) response, which is considered as a specific characteristic of cancer that enhances the growth, migration, and metastasis of tumors [9]. NF-κB is activated by intracellular and microenvironmental factors, such as TGF-β, promoting the differentiation of mesenchymal cells [10, 11], and then modulates a family of transcription factors and other proteins, including SNAIL, ZEB1 and TWIST1, that mediate the entire mesenchymal process, [9]. In addition, NF-κB can exert a direct promoting effect on the expression of multiple mesenchymal proteins. Regarding this point, it has been proven that the N-cadherin (CDH2) promoter contains an NF-κB binding site [12]. At the same time, Vimentin (VIM) is a typical NF-κB target gene [13], that is co-induced via p65 and relB to enhance mesenchymal differentiation [14, 15]. Thus, investigating the underlying mechanism of NF-κB-mediated EMT in the inflammatory microenvironment in glioma might provide novel strategies for glioma treatment.

ncRNAs (noncoding RNAs) have been implicated as critical players in the process of normal cells transforming into cancer cells. In recent years, numerous studies have demonstrated that two key types of regulatory ncRNAs, miRNAs (microRNAs) and lncRNAs (long noncoding RNAs), are strongly correlated with the EMT of glioma cells [16, 17]. Commonly, lncRNAs exert an effect on the expression of miRNA-targeted mRNAs via interaction with miRNAs. For example, Sox4 and H19 are considered the target genes of miR-130a-3p. The sponge effect of H19 on miR-130a-3p abolishes the inhibitory effect of miR-130a-3p on Sox4 [18], subsequently modulating EMT progression in glioma. Another lncRNA, FOXD2 AS1, can downregulate the expression of VIM and CDH2 and upregulate the expression of E-cadherin [19, 20], therefore inducing EMT in glioma. The inducing effect of FOXD2 AS1 on EMT depends strongly upon both FOXD2 AS1/miR-185-5p/cyclin D2 and FOXD2 AS1/miR-185/AKT1 axes [19, 20]. Interestingly, several studies have indicated that many lncRNAs and miRNAs deregulated in glioma [21–23], suggesting that other lncRNA/miRNA/mRNA axes might modulate glioma EMT, therefore affecting glioma progression.

Herein, we validated the lncRNAs and miRNAsthat might be related to NF-κB-mediated EMT in glioma cells based on the TCGA database, GEO microarray expression profiles, and previous literature. The predicted NF-κB regulation of lncRNA was first verified. The expression and specific functions of candidate lncRNAs and miRNAs, as well as the predicted binding between lncRNAs and miRNAs, were examined. Moreover, we also investigated possible downstream target mRNAs of miRNAs and the signaling pathways involved. Finally, the dynamic effects of the lncRNA/miRNA/mRNA axes on glioma cells were investigated. In summary, we propose novel axes formed by lncRNAs, miRNAs, and mRNAs that might modulate the EMT in glioma cells, therefore affecting glioma progression.

## RESULTS

### Selection of lncRNAs related to glioma overall survival

**Figure 1:**
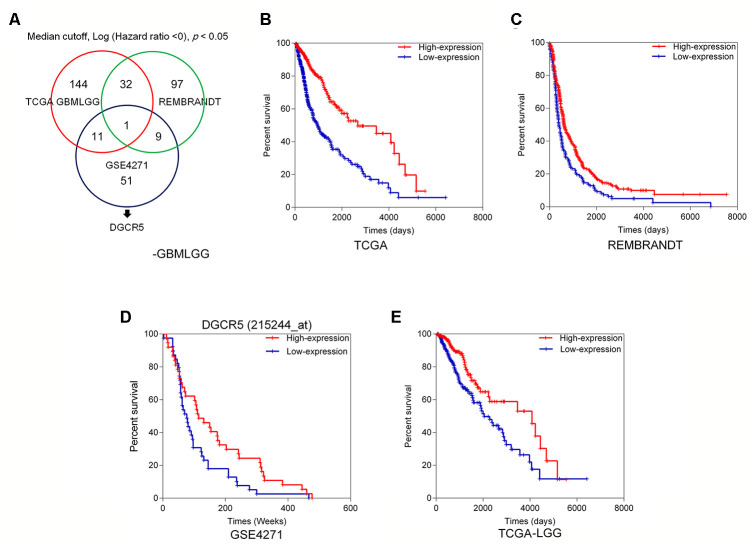
Selection of lncRNAs related to glioma overall survival (**A**) A schematic diagram showing the process of lncRNA selection. (**B**) Based on TCGA GBMLGG dataset, we selected 144 lncRNAs that are correlated with better overall survival in glioma patients when they are highly expressed. (**C**) Based on REMBRANDT dataset, 32 of the 144 lncRNAs are correlated with better overall survival in glioma patients when they are highly expressed. (**D**) Based on online microarray expression profile (GSE4271), higher lncRNA DGCR5 expression is significantly correlated with better overall survival in glioma patients. (**E**) Based on TCGA Brain Lower Grade Glioma (LGG) dataset, higher lncRNA DGCR5 expression is significantly correlated with better overall survival in glioma patients.

To investigate lncRNAs that might affect glioma EMT and progression, we first analyzed the correlation between deregulated lncRNAs and overall survival in glioma patients. Figure 1A shows the analysis and selection of candidate lncRNAs according to the TCGA GBMLGG dataset, REMBRANDT dataset, and microarray expression profiles from the GEO database. Based on the TCGA GBMLGG dataset, we selected 144 lncRNAs that were positively correlated with better overall survival in glioma patients when they were highly expressed (Figure 1B). Analysis of the REMBRANDT database, showed that 32 of the 144 lncRNAs had a positive correlation with better overall survival in patients with glioma when they were highly expressed (Figure 1C). Based on the online microarray expression profileGSE4271, higher lncRNA DGCR5 expression was significantly positively correlated with better overall survival in glioma patients (Figure 1D). Based on the TCGA Brain Lower Grade Glioma (LGG) dataset, higher lncRNA DGCR5 expression was significantly positively correlated with better overall survival in glioma patients (Figure 1E). According to all these online data, we hypothesized that lncRNA DGCR5 might play a protective role against glioma progression.

### Expression of lncRNA DGCR5 according to online data and experimental results

**Figure 2:**
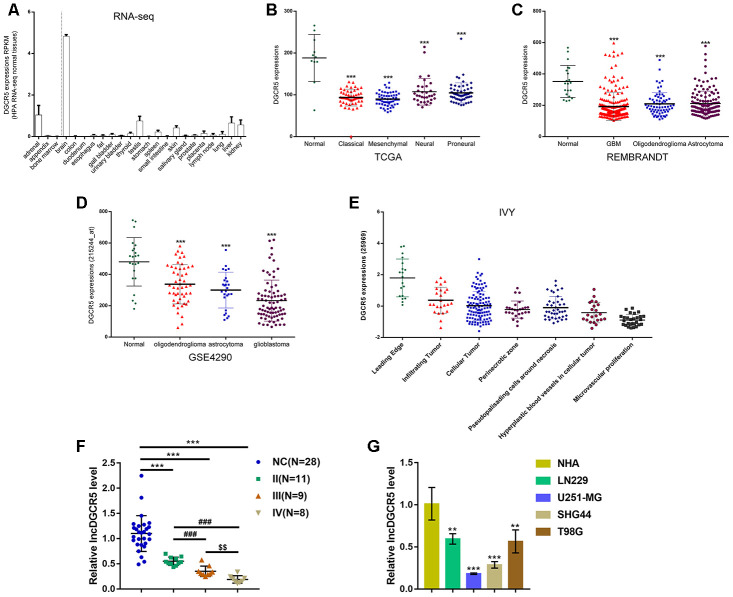
**Expression of lncRNA DGCR5 according to online data and experimental results.** (**A**) LncRNA DGCR5 expression in 27 types of tissues from 95 persons according to online HPA RNA-seq data. (**B**) LncRNA DGCR5 expression in normal (noncancerous), proneural glioma, neural glioma, mesenchymal glioma, and classical glioma according to TCGA dataset. (**C**) LncRNA DGCR5 expression in oligodendroglioma, astrocytoma, and glioblastoma according to REMBRANDT dataset. (**D**) LncRNA DGCR5 expression in oligodendroglioma, astrocytoma, and glioblastoma according to GSE4290. (**E**) LncRNA DGCR5 expression in different parts of glioma, including leading edge, infiltrating tumor, perinecrotic zone, cellular tumor, pseudopalisading cells around necrosis, hyperplastic blood vessels in cellular tumor, and microvascular proliferation according to Ivy Glioblastoma Atlas Project data. (**F**) LncRNA DGCR5 expression in normal (noncancerous, n=28) and glioma tissues (stage II n=11, stage III n=9, stage IV n=8) determined by real-time PCR. (**G**) LncRNA DGCR5 expression in normal human astrocytes, NHA, and glioma cell lines, LN229, U251-MG, SHG44, and T98G was determined by real-time PCR. **P<0.01, ***P<0.005, compared to control group. ###*P*<0.005, compared to stage II group. $$*P*<0.01, compared to stage III group.

To confirm this hypothesis, we examined lncRNA DGCR5 expression under different grouping conditions. According to online HPA RNA-seq data, DGCR5 expression was specifically upregulated in brain tissues among 27 types of tissues from 95 persons (Figure 2A). According to TCGA data, the expression of lncRNA DGCR5 was reduced in proneural glioma, neural glioma, mesenchymal glioma, and classical glioma, compared with noncancerous (normal) tissue samples (Figure 2B). According to REMBRANDT data, lncRNA DGCR5 expression was dramatically downregulated in oligodendroglioma, astrocytoma, and glioblastoma, compared with normal (noncancerous) tissues (Figure 2C). According to GSE4290, lncRNA DGCR5 expression showed to be remarkably downregulated within oligodendroglioma, astrocytoma, and glioblastoma, in comparison with that in normal (non-cancerous) tissues (Figure 2D). According to Ivy Glioblastoma Atlas Project data, lncRNA DGCR5 expression was analyzed in different parts of glioma, including leading-edge, infiltrating carcinoma, perinecrotic zone, cellular tumor, pseudoproliferative cells surrounding necrosis, proliferative blood vessels in cell tumor, and microvascular proliferation glioma. Figure 2E shows that lncRNA DGCR5 expression was the highest in the leading edge, whereas it was the lowest in microvascular proliferation glioma. Since microvascular proliferation glioma is the most malignant and invasive, these data indicated that lncRNA DGCR5 expression continued to be downregulated with tumor progression.

To further confirm this hypothesis, we examined lncRNA DGCR5 expression in tissue samples and cells. Figure 2F shows that the expression of lncRNA DGCR5 was significantly reduced in glioma tissue samples (stage II, n=11; stage III, n=9; stage IV, n=8), in comparison with normal (noncancerous, n=28) tissues. Moreover, DGCR5 expression was lower in stage III tissue samples and the lowest in stage IV tissues (Figure 2F), further suggesting that lncRNA DGCR5 expression continued to be downregulated with tumor progression. In cell lines, lncRNA DGCR5 expression was markedly downregulated in the four glioma cell lines, LN229, U251-MG, SHG44, and T98G compared to that in normal human astrocytes (NHA) (Figure 2G).

### NF-κB1 inhibits the expression of lncRNA DGCR5 by targeting its promoter region

As we have mentioned, NF-κB is considered a common transcriptional factor and exerts a significant effect on the mediation of various major characteristics related to mesenchymal differentiation [29]. According to TCGA data (Supplementary Figure 1A), GSE4290 (Supplementary Figure 1B), REMBRANDT data (Supplementary Figure 1C), CGGA data (Supplementary Figure 1D), and IVY data (Supplementary Figure 1E), NF-κB1 (p50) expression was negatively correlated with DGCR5 expression. Moreover, we performed Kyoto Encyclopedia of Genes and Genomes (KEGG) and Gene Set Enrichment Analysis (GSEA) on DGCR5 and found that DGCR5 was correlated with NF-κB1 signaling, including interferon α response and interferon γ response (Supplementary Figure 2A, 2B). More importantly, by using the online tool JASPER (http://jaspar.genereg.net/) according to the ensemble binding database, we found that the DGCR5 promoter region possesses NF-κB1 response element (RE) (Supplementary Table 1). Thus, we hypothesized that NF-κB1 can bind to the DGCR5 promoter region to inhibit its expression.

**Figure 3:**
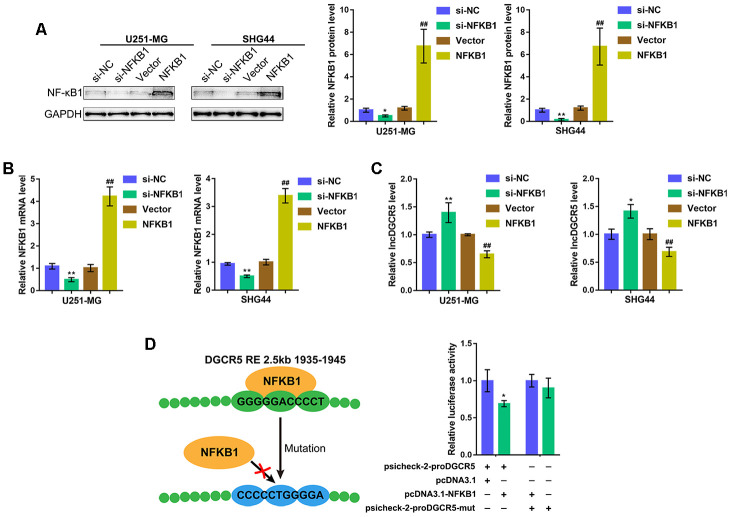
**NF-κB1 binds to the promoter region of lncRNA DGCR5 to inhibit its expression.** (**A**, **B**) NF-κB1 overexpression or silencing generated in U251-MG and SHG44 cells by transfection of NF-κB1-overexpressing vector or si-NF-κB1, as confirmed by immunoblotting and real-time PCR. (**C**) DGCR5 expression in response to NF-κB1 overexpression or silencing determined by real-time PCR. (**D**) Wild-type and mutant-type psicheck-2-proDGCR5 luciferase reporter vectors were constructed as described in the Materials and methods section. These vectors were cotransfected in 293T cells with pcDNA3.1/NF-κB1 and the luciferase activity was determined. *P<0.05, **P<0.01, compared to the si-NC group. ##*P*<0.01, compared to vector group.

To verify the hypothesis, we overexpressed or silenced NF-κB1 in U251-MG and SHG44 cells by transfection of an NF-κB1-overexpressing vector or si-NF-κB1 and performed immunoblotting and real-time PCR to verify the transfection efficiency (Figure 3A, 3B). Then, DGCR5 expression was determined in NF-κB1 or si-NF-κB1 transfected glioma cells. As expected, NF-κB1 overexpression significantly inhibited DGCR5 expression, whereas NF-κB1 silencing promoted DGCR5 expression (Figure 3C). To verify the predicted binding between NF-κB1 and the NF-κB1 RE in the DGCR5 promoter region, two different types of psiCHECK-2-proDGCR5 luciferase reporter vectors, wild-type and mutant, were constructed as described (Figure 3D). We cotransfected these vectors into 293T cells with pcDNA3.1/NF-κB1 and examined luciferase activity. Figure 3D shows that psiCHECK-2-proDGCR5 luciferase activity was significantly decreased via the overexpression of NF-κB1. Mutating the predicted NF-κB1 RE in the DGCR5 promoter region abolished the alterations in luciferase activity (Figure 3D). Therefore, considering that NF-κB1 exerts an essential effect on the mediation of various major characteristics related to mesenchymal differentiation [29], we hypothesize that DGCR5 exerts an effect on mesenchymal differentiation] in glioma.

### Effects of lncRNA DGCR5 overexpression on glioma

To validate the specific effect of lncRNA DGCR5 on glioma cells, we first constructed the coexpression network consisting of DGCR5 and its negatively-correlated genes according to TCGA data, REMBRANDT data, and GSE4290. As shown in Supplementary Figure 3A and Supplementary Table 2, a total of 18 genes were significantly negatively correlated with DGCR5, including GNAI3 [30], VIM [31], ITGB1 [32, 33], HIF1A [34], and HDAC1 [35], all of which are critical factors affecting glioma development. The expression of these five genes was significantly upregulated in U251-MG and SHG44 cells (Supplementary Figure 3B–3F). Thus, further experiments were performed using U251-MG and SHG44 cells.

**Figure 4:**
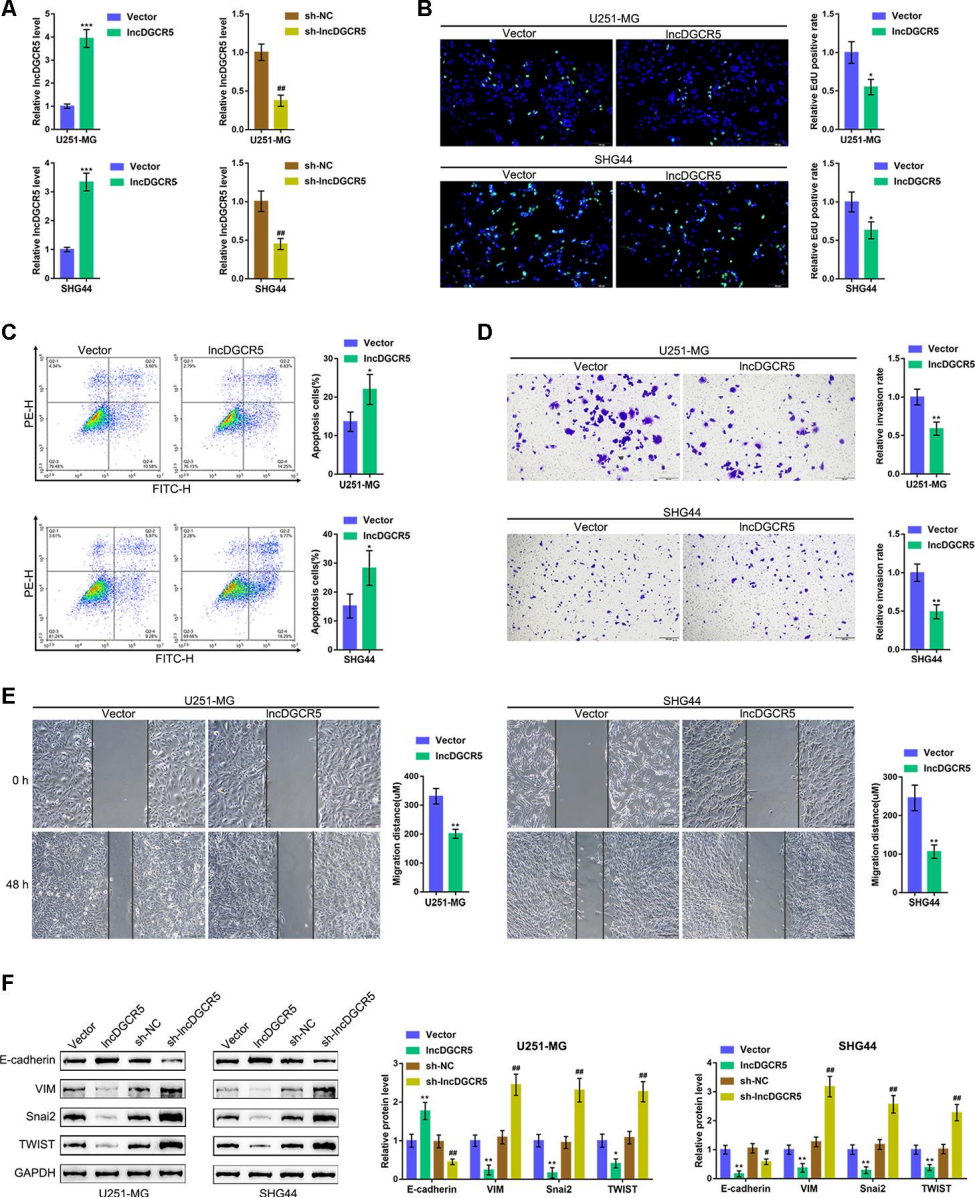
**Effects of lncRNA DGCR5 overexpression on glioma cells.** (**A**) LncRNA DGCR5 overexpression or silencing was generated in U251-MG and SHG44 cells by transfection of lncRNA DGCR5-overexpressing vector (lncDGCR5) or small hairpin RNA (sh-DGCR5), as confirmed by real-time PCR. Next, DNA synthesis capacity was determined by EdU assay (**B**); cell apoptosis was determined by Flow cytometry assay (**C**); cell invasive capacity was determined by Transwell assay (**D**); cell migratory capacity was determined by Wound healing assay (**E**). (**F**) U251-MG and SHG44 cells were transfected with lncDGCR5 or sh-DGCR5 and the protein levels of E-cadherin, VIM, Snai2, and TWIST were determined by immunoblotting. *P<0.05, **P<0.01, ***P<0.005 compared to vector group; ## *P*<0.01 compared to sh-NC group.

Next, we overexpressed and silenced lncRNA DGCR5 in U251-MG and SHG44 cells by transfection of the lncRNA DGCR5-overexpressing vector or short hairpin RNA (sh-DGCR5) and performed real-time PCR to verify the transfection efficiency (Figure 4A). Next, we examined the specific effects on glioma cells. LncRNA DGCR5 overexpression significantly inhibited the capacity of DNA synthesis (Figure 4B), invasion (Figure 4D) and migration (Figure 4E) in glioma cells, whereas it enhanced the apoptosis rate of glioma cells (Figure 4C). Regarding the signaling involved, the overexpression of lncRNA DGCR5 dramatically enhanced the protein levels of E-cadherin but inhibited the protein levels of VIM, Snai2, and TWIST (Figure 4F); in contrast, DGCR5 silencing significantly decreased E-cadherin protein but increased VIM, Snai2, and TWIST proteins (Figure 4F). In summary, lncRNA DGCR5 might inhibit the capacity of glioma cells to proliferate, migrate, and invade, thus exerting a tumor-suppressive effect.

### LncRNA DGCR5 directly binds to miR-21-3p and miR-23a-5p

**Figure 5:**
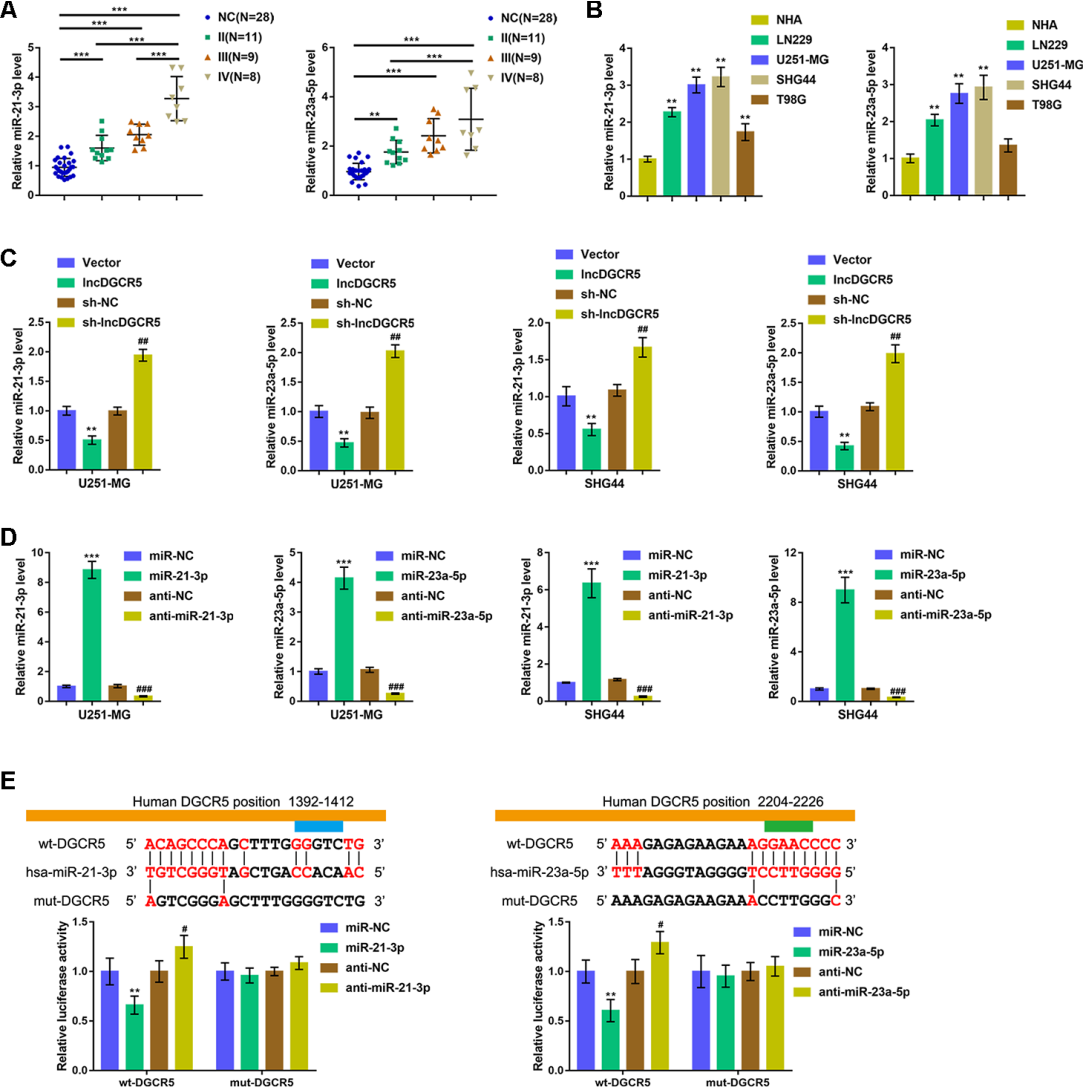
LncRNA DGCR5 directly binds to miR-21-3p and miR-23a-5p (**A**) miR-21-3p and miR-23a-5p expression was determined in normal (non-cancerous, n=28) and glioma tissues (stage II n=11, stage III n=9, stage IV n=8) by real-time PCR. (**B**) miR-21-3p and miR-23a-5p expression in normal human astrocytes, NHA, and glioma cell lines, LN229, U251-MG, SHG44, and T98G was determined by real-time PCR. (**C**) U251-MG and SHG44 cells were transfected with lncDGCR5 or sh-DGCR5 and the expression of miR-21-3p and miR-23a-5p was determined by real-time PCR. (**D**) miR-21-3p and miR-23a-5p overexpression or inhibition generated in U251-MG and SHG44 cells by transfection of miR-21-3p/miR-23a-5p or anti-miR-21-3p/anti-miR-23a-5p, as confirmed by real-time PCR. (**E**) Wild-type and mutant-type lncRNA DGCR5 luciferase reporter vectors were constructed as described in Materials and methods section. These vectors were co-transfected in 293T cells with miR-21-3p/miR-23a-5p or anti-miR-21-3p/anti-miR-23a-5p, and the luciferase activity was determined. **P<0.01, ***P<0.005, compared to NHA, vector or miR-NC group; #*P*<0.05, ##*P*<0.01, ###*P*<0.005, compared to sh-NC or anti-NC group.

Commonly, lncRNAs exert an effect on the expression of miRNA-targeted mRNA via interaction with miRNAs. To further investigate the suppressive effects of lncRNA DGCR5 on the proliferation, migration, and invasion of glioma cells, we analyzed TCGA data to identify miRNAs that were negatively correlated with DGCR5. As shown in Supplementary Figure 4, miR-21 and miR-23a were negatively correlated with DGCR5. Reportedly, miR-21 is regarded as promising treatment target for glioma [36]. In addition, miR-23a is also implicated in promoting glioma cell proliferation [37]. In tissue samples, the expression of miR-21/miR-23a was significantly upregulated in glioma tissue samples (stage II, n=11; stage III, n=9; stage IV, n=8) compared with normal (noncancerous, n=28) tissues. Moreover, miR-21/miR-23a expression was higher in stage III tissue samples and highest in stage IV tissues (Figure 5A). Accordingly, the expression of miR-21/miR-23a was significantly upregulated in glioma cell lines compared with NHAs (Figure 5B). Moreover, online tools predicted that miR-21 and miR-23a might both be the downstream targets of DGCR5. To confirm that lncRNA DGCR5 negatively regulates miR-21/miR-23a, we transfected U251-MG and SHG44 cells with lncDGCR5 or sh-DGCR5 and examined the expression levels of miR-21/miR-23a. As shown in Figure 5C, lncRNA DGCR5 overexpression significantly downregulated, whereas lncRNA DGCR5 silencing significantly upregulated the expression levels of miR-21/miR-23a. Thus, we hypothesized that DGCR5 exerts its tumor-suppressive effects through miR-21/miR-23a.

To verify this hypothesis, we transfected U251-MG and SHG44 cells with miR-21-3p/miR-23a-5p or anti-miR-21-3p/anti-miR-23a-5p to achieve overexpression or inhibition of miR-21-3p and miR-23a-5p and performed real-time PCR to verify the transfection efficiency (Figure 5D). Second, we conducted a luciferase reporter assay and constructed two different types of lncRNA DGCR5 luciferase reporter vectors, wild-type and mutant, as described, to verify the putative binding sites. We cotransfected these vectors into 293T cells with miR-21-3p/miR-23a-5p or anti-miR-21-3p/anti-miR-23a-5p, and examined luciferase activity. Figure 5E shows that wild-type DGCR5 luciferase activity was suppressed via the overexpression of miR-21/miR-23a, but enhanced via the inhibition of miR-21/miR-23a. Mutating the putative DGCR5 bindings sites abolished alterations in the luciferase activity (Figure 5E). These data indicate that miR-21 and miR-23a are downstream targets of lncRNA DGCR5.

### The DGCR5/miR-21 axis affects glioma cell migration and invasion through Smad7

**Figure 6:**
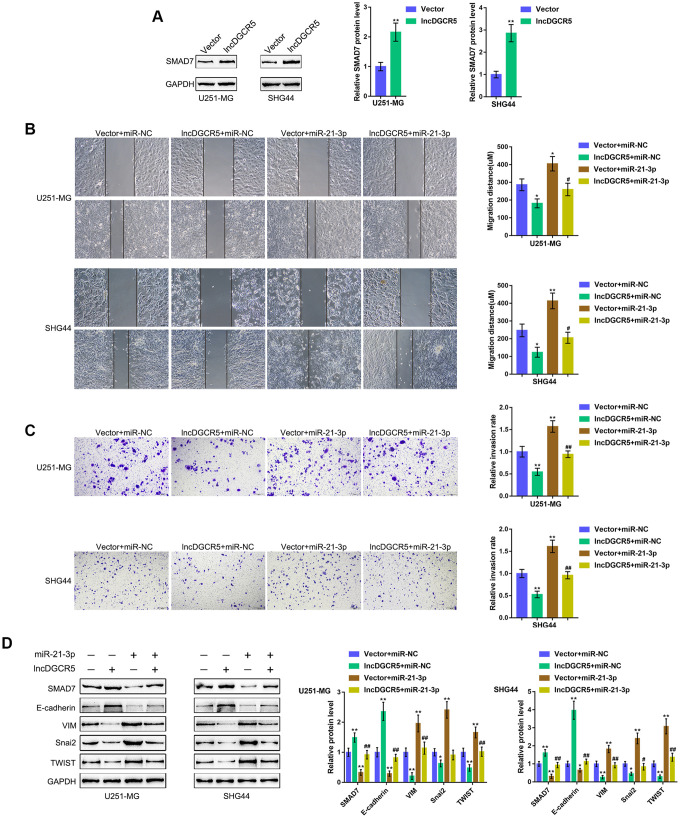
**DGCR5/miR-21 axis affect glioma cell migration and invasion through Smad7.** (**A**) U251-MG and SHG44 cells were transfected with lncRNA DGCR5-overexpressing vector and examined for the protein levels of Smad7 by immunoblotting. Next, U251-MG and SHG44 cells were co-transfected with DGCR5 and miR-21 mimic and examined for (**B**) migratory capacity by wound healing assay; (**C**) invasive capacity by Transwell assay; (**D**) the protein levels of Smad7, E-cadherin, VIM, Snai2, and TWIST were determined by immunoblotting. *P<0.05, **P<0.01, compared to the control group; #*P*<0.05, ##*P*<0.01, compared to the vector + miR-21 mimic group.

The effect of the miR-21/Smad7 axis has been found in many diseases [38–40]. Smad7 is a TGFβ1 signaling inhibitor and has been reported to inhibit TGFβ1-induced cancer cell epithelial-mesenchymal transition (EMT) and metastasis [41–43]. In the present study, Smad7 was identified as a direct downstream target of miR-21; thus, the cellular functions of miR-21 might be related to cancer cell migration and invasion. Moreover, online data also showed that DGCR5 expression was significantly positively correlated with Smad7 expression (Supplementary Figure 5A–5E). Thus, we investigated whether the DGCR5/miR-21 axis affects Smad7 to act on glioma cell migration and invasion. We transfected U251-MG and SHG44 cells with lncRNA DGCR5-overexpressing vector and examined Smad7 protein levels. Figure 6A shows that the overexpression of lncRNA DGCR5 remarkably increased Smad7 protein levels in both U251-MG and SHG44 cell lines. Second, we cotransfected U251-MG and SHG44 cells with DGCR5 and miR-21 mimic and evaluated their dynamic effects on glioma cells. In contrast to DGCR5 overexpression, the overexpression of miR-21 dramatically promoted the capacity of glioma cells to migrate and invade (Figure 6B, 6C). Moreover, the overexpression of miR-21 significantly attenuated the effects of DGCR5 overexpression (Figure 6B, 6C). Regarding the signaling involved, miR-21 overexpression significantly decreased Smad7 and E-cadherin proteins, but increased VIM, Snai2, and TWIST proteins (Figure 6D). Accordingly, the overexpression of miR-21 significantly attenuated the effects of DGCR5 overexpression on these protein levels (Figure 6D). In summary, the DGCR5/miR-21 axis affects glioma cell migration, invasion, and EMT through Smad7.

### The DGCR5/miR-23a axis affects glioma cell proliferation and apoptosis through PTEN

**Figure 7:**
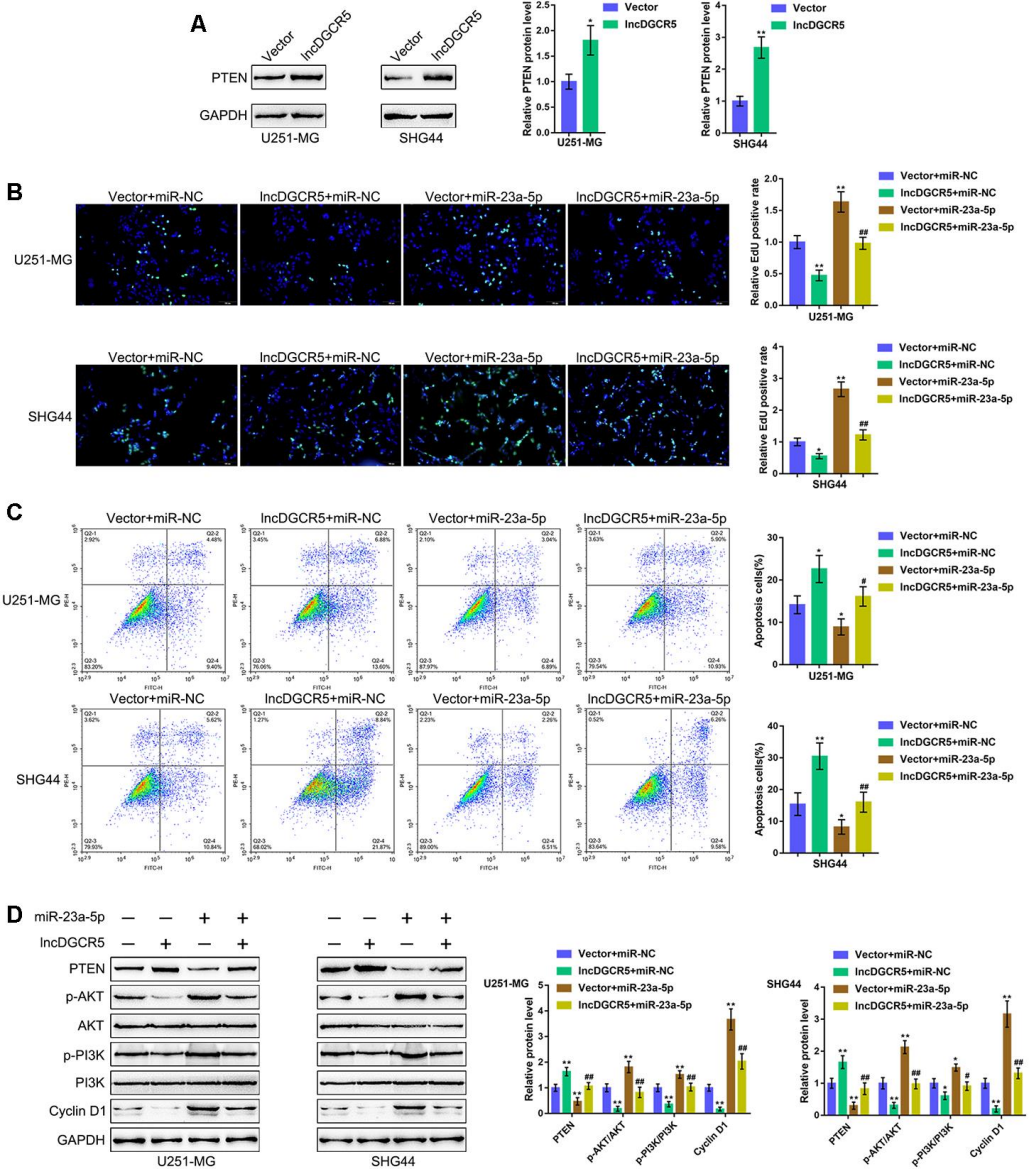
**DGCR5/miR-23a axis affect glioma cell proliferation and apoptosis through PTEN.** (**A**) U251-MG and SHG44 cells were transfected with lncRNA DGCR5-overexpressing vector and examined for the protein levels of PTEN by immunoblotting. Next, U251-MG and SHG44 cells were co-transfected with DGCR5 and miR-23a mimic, (**B**) DNA synthesis capacity was determined by EdU assay (**C**); cell apoptosis was determined by Flow cytometry assay; (**D**) the protein levels of PTEN, p-AKT, AKT, p-PI3K, PI3K, and Cyclin D1 were determined by immunoblotting. *P<0.05, **P<0.01, compared to the control group; #*P*<0.05, ##*P*<0.01, compared to the vector + miR-23a mimic group.

PTEN is often knocked down in glioma cells and has been identified as a miR-23a downstream target in glioma [44]. Similarly, online data demonstrated a significant positive correlation between DGCR5 expression and PTEN expression (Supplementary Figure 6A–6D). The tumor-suppressor activity of PTEN depends largely on its lipid phosphatase activity, which inhibits PI3K/AKT activation. Activated AKT has many downstream effects, including the promotion of cell proliferation, increase in cellular glycolytic flux, and inhibition of apoptosis. PTEN antagonizes this survival/proliferative paradigm [45, 46]. In the present study, PTEN was identified as a direct downstream target of miR-23a; thus, the cellular functions of miR-23a might be related to cancer cell survival/proliferation/apoptosis. Therefore, we speculated that the DGCR5/miR-23a axis might affect PTEN to act on glioma cell proliferation and apoptosis. To verify this hypothesis, we transfected U251-MG and SHG44 cells with the lncRNA DGCR5-overexpressing vector and evaluated PTEN proteins. As shown in Figure 7A, DGCR5 overexpression markedly enhanced PTEN protein levels in the two glioma cell lines. Second, we cotransfected U251-MG and SHG44 cells with DGCR5 and miR-23a mimic and evaluated the dynamic effects of DGCR5 and miR-23a overexpression. miR-23a overexpression significantly increased glioma cell proliferation, and enhanced glioma cell apoptosis (Figure 7B, 7C). Moreover, the overexpression of miR-23a dramatically attenuated the effects of DGCR5 overexpression (Figure 7B, 7C). Regarding the signaling involved, miR-23a overexpression significantly decreased the protein levels of PTEN, increased AKT and PI3K phosphorylation and increased Cyclin D1 protein levels (Figure 7D). Similarly, the overexpression of miR-23a significantly attenuated the effects of DGCR5 overexpression on these protein levels (Figure 7D). In summary, the DGCR5/miR-23a axis affects glioma cell proliferation and apoptosis through PTEN/PI3K/AKT.

### *In vivo* effects of lncRNA DGCR5 on tumor growth

**Figure 8:**
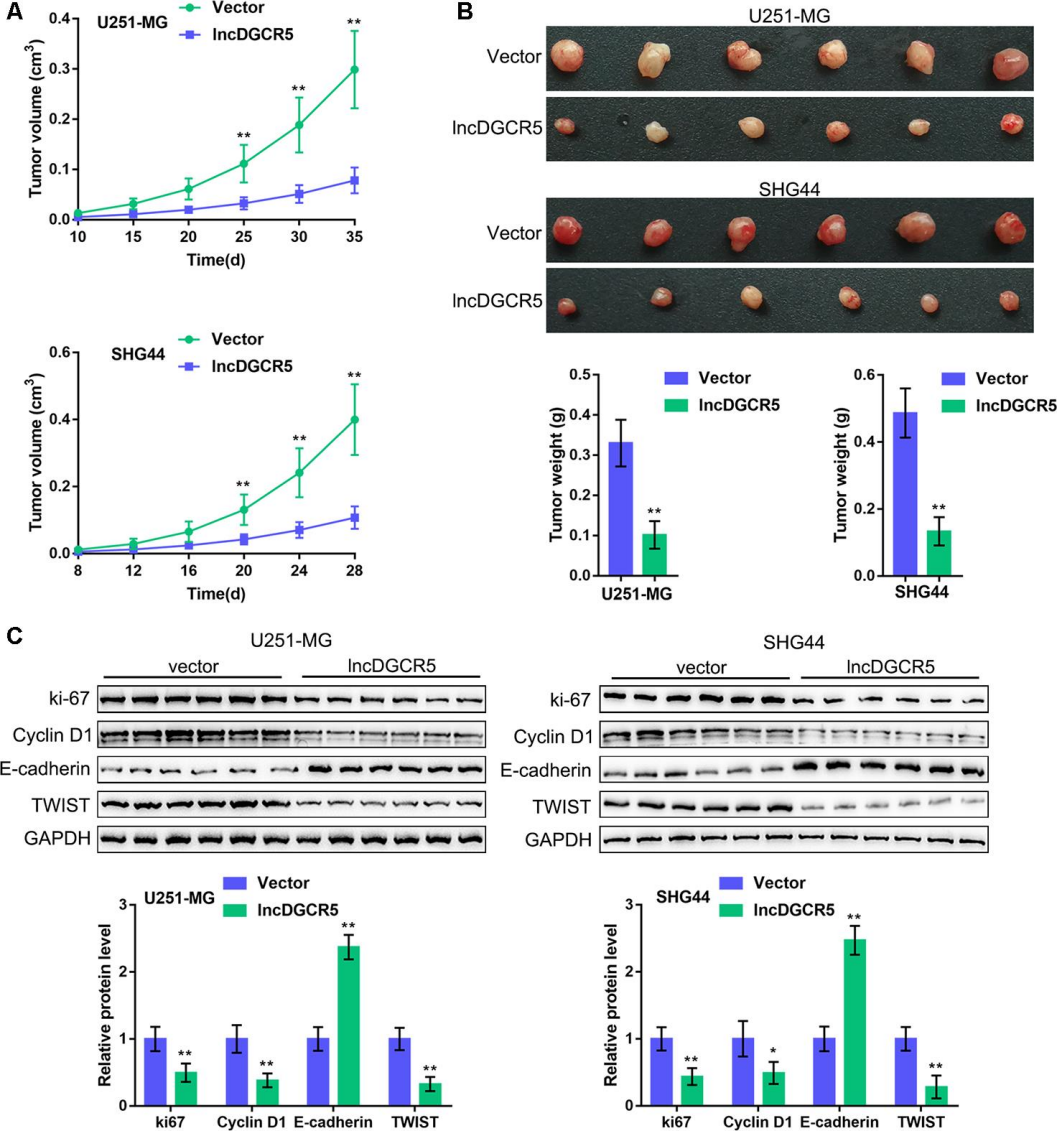
***In vivo* effects of lncRNA DGCR5 on tumor growth An xenograft model was established in mice as described in the Materials and methods section.** (**A**, **B**) The tumor volume and weight were examined after the mice were sacrificed. (**C**) Tumor tissues were collected and the protein levels of ki-67, Cyclin D1, TWIST, and E-cadherin were determined by immunoblotting. *P<0.05, **P<0.01.

To further confirm these *in vitro* findings, we established an orthotopic implanted xenograft mouse model. We examined the effects of lncRNA DGCR5 on tumor growth, proliferation markers, cell cycle regulators, and EMT markers. As shown in Figure 8A–8B, lncRNA DGCR5 overexpression significantly reduced the tumor volume and weight of both U251-MG-derived and SHG44-derived tumors. Moreover, DGCR5 overexpression significantly decreased the protein levels of proliferation marker ki-67, cell cycle regulator Cyclin D1, and mesenchymal marker TWIST, but increased the protein level of the epithelial marker E-cadherin in tumor tissues (Figure 8C). These data indicate that lncRNA DGCR5 acts as a tumor-suppressor to inhibit glioma tumor growth *in vivo*.

## DISCUSSION

Under the inflammatory microenvironment, NF-κB1-mediated EMT is considered to be a critical event in the development of glioma. We identified lncRNA DGCR5 as a downregulated lncRNA in glioma that could be negatively regulated by NF-κB1 through direct binding. LncRNA DGCR5 overexpression significantly inhibited the capacity of glioma cells to proliferate, migrate, and invade, whereas promoted the apoptosis of glioma cells. Moreover, lncRNA DGCR5 overexpression upregulated the epithelial marker E-cadherin while downregulating the mesenchymal marker VIM, as well as Snai2 and TWIST. Regarding the underlying molecular mechanisms, lncRNA DGCR5 could target to inhibit miR-21 and miR-23a expression, and miR-21 or miR-23a overexpression significantly reversed the tumor-suppressive effects of lncRNA DGCR5 overexpression. LncRNA DGCR5 exerted its tumor-suppressive effects through the DGCR5/miR-21/Smad7 and DGCR5/miR-23a/PTEN axes. *In vivo* experiments in an orthotopic implanted xenograft model further confirmed that lncRNA DGCR5 acts as a tumor-suppressor to inhibit glioma tumor growth.

The primary NF-κB dimer found in stationary glioblastoma cells is composed of p50 (NF-κB1) and p65 [47, 48]. NF-κB has long been regarded as an essential regulator of TGF-β-elicited EMT in breast epithelial cells that overexpress the Ras oncogene [49]. The blockage of NF-κB suppressed EMT transformation, whereas forced NF-κB overexpression induced EMT in a TGF-β-independent manner. Moreover, knocking down NF-κB in mesenchymal cells could restore the epithelial phenotype. Another group reported that NF-κB exerts a positive regulatory effect on Snail expression, and is suppressed via upstream GS3, which acts as the essential mediatory factor for both epithelium maintenance and the protein levels of E-cadherin [50, 51]. Herein, we found that NF-κB1 inhibited the expression of NF-κB1 RE in glioma cells via direct binding to the NF-κB1 RE in the lncRNA DGCR5 promoter region. According to a series of online data, lncRNA DGCR5 expression was found to be significantly downregulated in glioma, especially in samples from advanced stages. Thus, it is reasonable to hypothesize that lncRNA DGCR5 might play a role in glioma progression, possibly by regulating EMT.

LncRNA DGCR5 acts as a tumor-suppressor lncRNA in a variety of cancers. In lung cancer, DGCR5 bound to miR-1180 to suppress AKT, GSK-3β, and β-catenin expression, therefore suppressing the capacity of H520 and H1299 lung cancer cells to proliferate, migrate and invade [52]. In gastric cancer, lncRNA DGCR5 is known for its function as a ceRNA (competing endogenous RNA) of PTEN and BTG1 through miR-23b to inhibit the capacity of gastric cancer cells to proliferate, invade, and migrate while promoting the apoptosis of these cells [53]. Similarly, overexpression of lncRNA DGCR5 in U251-MG and SHG44 cells dramatically increased the capacity of glioma cells to proliferate, migrate, and invade, suggesting that lncRNA DGCR5 exerted a tumor-suppressive effect on glioma. Regarding the molecular mechanism, DGCR5 overexpression significantly increased epithelial marker E-cadherin protein levels while reducing the protein levels of mesenchymal marker VIM, as well as Snai2 and TWIST. Snai2 is associated with tumor development and aggressiveness by changing the gene expression of E-cadherin and VIM [54, 55], and functions as a direct inhibitor of E-cadherin by targeting the specific E-boxes of the E-cadherin proximal promoter [56]. Twist is also regarded as an essential regulatory factor of EMT *in vitro* and *in vivo* in metastatic and aggressive tumors [57] by causing transcriptional repression of E-cadherin. Based on these findings, lncRNA DGCR5 was demonstrated to block EMT in glioma cells, thereby acting as a tumor suppressor.

LncRNAs can play a pivotal role in the regulation of gene expression in a variety of ways, including chromosome remodeling and processing at the transcriptional and posttranscriptional levels [58], such as acting as competitive endogenous RNAs (ceRNAs). Noncoding RNAs do not participate in the translation of active proteins, so they are more efficient than protein-coding RNAs in miRNA binding [59]. Herein, miR-21 and miR-23a, two significantly increased miRNAs in glioma, were both predicted to be downstream targets of lncRNA DGCR5. In glioma cell lines, DGCR5 negatively regulated the expression levels of miR-21 and miR-23a. Thus, DGCR5 might exert its effects on glioma cells through miR-21 and miR-23a and their respective targets.

miR-21 could enhance TGF-β-elicited EMT by binding to Smad7 [40, 60]. As we have mentioned, Smad7 is a TGFβ1 signaling inhibitor and has been reported to inhibit TGFβ1-induced cancer cell EMT and metastasis [41–43]. Although Smad7 has a dual role in carcinogenesis and cancer cell growth and its functions depend on the type of cancer [61], in glioma, it has been reported to act as a tumor suppressor. TGFβ enhances the expression of USP26 and reinforces SMAD7 stability by limiting the ubiquitin-mediated turnover of SMAD7. Conversely, knockdown of USP26 rapidly degrades SMAD7 resulting in TGFβ receptor stabilization and enhanced levels of p-SMAD2, ultimately enhancing cellular proliferation, invasion, and metastasis [62]. Another study identified SMAD7 as a target of miR-195 in glioma and showed that TGFβ1 promotes miR-195 expression, inhibits SMAD7 expression, and promotes U87 cell proliferation and invasion [63]. In the present study, we found that miR-21 directly binds to SMAD7 to inhibit SMAD7 expression. miR-21 overexpression promoted the capacity of glioma cells to migrate and invade and increased the epithelial marker E-cadherin protein, while decreasing the mesenchymal markers VIM, Snai2, and TWIST. These data are consistent with previous studies on SMAD7 functions in glioma.

**Figure 9:**
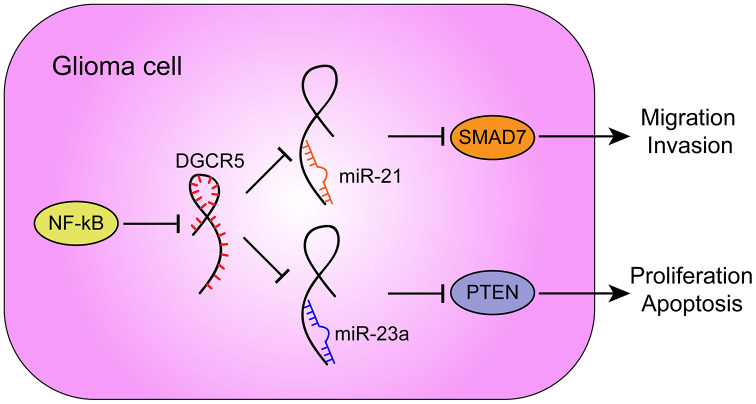
**A schematic diagram showing the roles and mechanisms of NF-κB/lncRNA DGCR5/miR-21/Smad7 and NF-κB/lncRNA DGCR5/miR-23a/PTEN in glioma carcinogenesis.**

Furthermore, -23a has been reported to target PTEN to activate AKT/ERK pathways and EMT in osteosarcoma cells [64]. miR-23a interacts with and degrades PTEN to further influence the downstream pathway PI3K/AKT/mTOR/Snail in hepatic fibrosis [65]. In the present study, miR-23a overexpression also played an oncogenic role. miR-23a overexpression promoted the proliferation and inhibited the apoptosis of glioma cells, as well as increased AKT and PI3K phosphorylation and Cyclin D1 protein levels. More importantly, miR-21 or miR-23a overexpression significantly attenuated the involvement of lncRNA DGCR5 overexpression in the capacity of glioma cells to proliferate, migrate, invade, and/or undergo apoptosis in these cells. In summary, lncRNA DGCR5 acts as a tumor suppressor through the DGCR5/miR-21/Smad7 and DGCR5/miR-23a/PTEN axes (Figure 9).

In conclusion, lncRNA DGCR5 suppresses the capacity of glioma cells to migrate and to invade via miR-21/Smad7, and it inhibits the proliferation and enhances the apoptosis of glioma cells through miR-23a/PTEN.

## MATERIALS AND METHODS

### Clinical tissue sampling

A total of 28 noncancerous peritumoral brain edema (PTBE) tissues and 28 glioma tissues (11 stage II, 9 stage III, 8 stage IV) were collected as described in our previous studies [24, 25] with the approval of Sichuan Provincial People’s Hospital. Sampling was obtained with singed informed consent from all participants. Once collected, samples were stored in liquid nitrogen or fixed in 4% paraformaldehyde.

### Cell lines and cell transfection

The human glioma cell lines LN229 (ATCC® CRL-2611) and T98G (ATCC® CRL-1690) were obtained from the American Type Culture Collection (ATCC, USA). SHG44 cells were obtained from the Cell Resource Center, Shanghai Institute of Life Sciences (Shanghai, China). U251-MG cells were purchased from the China Center for Type Culture Collection (CCTCC, Wuhan, China). All glioma cells were cultured in supplemented RPMI-1640 medium (Invitrogen, USA) supplemented with 10% fetal bovine serum (FBS; Gibco, Waltham, MA, USA) at 37 °C with 5% CO_2_. Normal human astrocytes (NHAs) were obtained from Lonza (Basel, Switzerland) and cultured in astrocyte basal medium (Lonza) supplemented with 10% FBS at 37 °C with 5% CO_2_.

The lncDGCR5-overexpressing vector (lncDGCR5) was used to generate DGCR5-overexpressing cells (GenePharma, Shanghai, China). The overexpression and inhibition of miR-21-3p and miR-23a-5p was achieved by transfection of miR-21-3p/miR-23a-5p or anti-miR-21-3p/anti-miR-23a-5p vectors (GenePharma). All transfections were performed with the help of Lipofectamine 3000 (Invitrogen, Waltham, MA, USA).

### PCR-based analyses

After extracting total RNA from target tissues or cells, we examined the expression levels of miRNAs and mRNAs using an SYBR Green PCR kit (Qiagen, Germany‎). The expression of RNU6B and GAPDH served as endogenous controls, for the determination of miRNA and mRNA expression, respectively. The data were processed and calculated using the 2^-ΔΔCT^ method.

### DNA synthesis capacity detected by the EdU assay

DNA synthesis was detected using EdU reagent following previously described methods [26]. Apollo staining and DAPI staining were used to examine the EdU positive cells with a fluorescence microscope following previously described methods [26].

### Cell apoptosis detected by flow cytometry

For apoptosis analysis, the quantification of apoptotic cells was performed with an Annexin V-FITC apoptosis detection kit (Keygen, Nanjing, China) following the previously described methods [27]. Propidium iodide (PI) was used for nuclear staining. The excitation wavelength and the emission wavelength were 488 nm and 530 nm, respectively.

### Migratory capacity detected by the wound healing assay

The cells were digested, and the cell concentration was adjusted to 5 × 10^5^ cells/ml. Cell suspensions (100 μl) were plated in 96-well plates coated with Matrigel, routinely cultured until the cell monolayer was established. Then the cell scratch test was performed. The cells were cultured in RPMI-1640 supplemented with 10 g/l bovine serum albumin (BSA) and 1% FCS, and the scratch area was measured under a microscope. Twenty-four hours later, the cells continued to culture for another 24 h in RPMI-1640 supplemented with 10% FCS. Then the relative distance of cell migration to the injury area was also measured under a microscope.

### Invasive capacity detected by the transwell assay

The invasive capacity was determined using polycarbonate Transwell filters following previously described methods [28]. Medium without serum was used in the upper chambers coated with Matrigel, and medium with serum was used in the bottom chambers. After discarding the noninvasive cells in the top chambers, the invasive cells on the lower membrane surface were fixed, stained with DAPI (Beyotime Institute of Biotechnology, Haimen, China) for nuclear staining, and the cell number was counted under a microscope.

### Protein expression levels detected by immunoblotting

Protein levels were determined following previously described methods [28] with the following antibodies: anti-E-cadherin (ab1416), anti-Vimentin (ab92547), anti-Snai2 (AV33302, Sigma-Aldrich, St. Louis, MO, USA), anti-TWIST (ab50887), anti-Smad7 (ab90086), anti-PTEN (ab32199), anti-AKT (ab179463), anti-p-AKT (ab81283), anti-PI3K (ab151549), anti-p-PI3K (ab182651), anti-Cyclin D1 (ab16663), anti-NF-κB1 (ZRB2082, Sigma-Aldrich), anti-GAPDH (ab8245), and Horseradish peroxidase-conjugated secondary antibody IgG (ab6721 and ab205719). Enhanced Chemiluminescent (ECL) Substrates (Millipore, MA, USA) were used for signals’ visualization taking Tubulin as an endogenous normalization control.

### Luciferase reporter assay

The fragment of lncRNA DGCR5 was amplified by PCR, cloned downstream of the Renilla gene in the psiCHECK-2 vector (Promega, Madison, WI, USA), and the resulting construct was named wt-DGCR5. To generate the DGCR5 mutant reporter, we mutated the seed region of DGCR5 to remove all complementarity to miR-21-3p and miR-23a-5p, and the resulting construct was named mut-DGCR5. These vectors were cotransfected into 293T cells with miR-21-3p/miR-23a-5p or anti-miR-21-3p/anti-miR-23a-5p. Luciferase assays were performed 48 h after transfection using the Dual-Luciferase Reporter Assay System (Promega). Renilla luciferase activity was normalized to firefly luciferase activity for each transfected well.

To investigate the binding of NF-κB1 to the DGCR5 promoter harboring the predicted NF-κB1 binding site, we cloned the DGCR5 promoter into psiCHECK-2 (Promega) to construct a DGCR5 promoter-containing luciferase reporter vector (psiCHECK-2-proDGCR5). Cells were cotransfected with these vectors and pcDNA3.1-NF-κB1 or blank pcDNA3.1. Luciferase assays were performed 48 h after transfection to determine the activities of firefly luciferase and Renilla luciferase.

### Xenograft model

To establish a human glioma cell xenograft nude mouse model, 24 female nude mice (BALB/c-nu/nu) at 6-week-old and weighing 16-20 g were obtained from the SLAC Laboratory Animal Center. A total of 24 mice were randomly divided into 4 groups: U251-MG +vector group, U251-MG +lncDGCR5 group, SHG44 +vector group, SHG44 +lncDGCR5 group. A total of 1x10^6^ transfected U251-MG or SHG44 cells were suspended in 100 μl PBS and subcutaneously injected into the right anterior armpits of the nude mice. Thirty-five days later, the animals were sacrificed and tumor weight and volume were examined and calculated. The tumor tissues were collected for protein level examination using immunoblotting. All experiments were repeated at least in triplicate. All animal experiments were performed with the approval of the ethics committee of the Sichuan Provincial People’s Hospital.

### Statistical analysis

The results from at least three independent experiments were processed using GraphPad and then expressed as the means ± SD. Data were statistically analyzed by one-way analysis of variance (ANOVA) followed by Tukey's multiple comparison test or independent sample *t*-test. A *P*-value of < 0.05 was considered significantly different.

## Supplementary Material

Supplementary Tables

Supplementary Figures
