# Supplementary material for: LncRNA DGCR5 plays a tumor-suppressive role in glioma via the miR-21/Smad7 and miR-23a/PTEN axes
Source: Aging (Albany NY). 2020 Oct 21;12(20):20285–307. doi: 10.18632/aging.103800 (PMC7655220; doi:10.18632/aging.103800)
Supplement: Supplementary Tables [file aging-12-103800-s001..pdf]

SUPPLEMENTARY TABLES

Supplementary Table 1. JASPAR predicts that NFKB1 binds to the upstream 2.5kb promoter element RE (ensemble binding database) of DGCR5.

Matrix ID	Name	Score	Relative score	Sequence ID	Start	End	Strand	Predicted sequence
MA0105.2	NFKB1	10.9251	0.863738922	3	1935	1945	+	GGGGGACCCCT
MA0105.2	NFKB1	10.7127	0.858678263	3	1935	1945	-	AGGGGTCCCCC
MA0105.2	NFKB1	9.54881	0.830954487	3	1760	1770	-	GGGGGTGTTCC
MA0105.2	NFKB1	8.96001	0.816928923	3	1624	1634	+	GGGGAGGACCC
MA0105.2	NFKB1	8.56588	0.807540503	3	939	949	-	GGGGAACCTCA
MA0105.3	NFKB1	7.6742	0.860929072	3	939	949	+	TGAGGTTCCCC

Supplementary Table 2. Negatively correlated genes of DGCR5 in various datasets of brain tumors.

	TCGA	GSE4290	REMBRANDT
SYDE1	-0.620	-0.662 (216272_x_at)	-0.325
TGIF1	-0.613	-0.673 (203313_s_at)	-0.610
GNAI3	-0.606	-0.676 (201179_s_at)	-0.658
ITGB1	-0.628	-0.647 (211945_s_at)	-0.559
VIM	-0.620	-0.654 (201426_s_at)	ND
DDOST	-0.622	-0.648 (208675_s_at)	-0.570
GPX7	-0.615	-0.661 (213170_at)	-0.602
PYGL	-0.601	-0.690 (202990_at)	-0.513
CLIC4	-0.601	-0.604 (201560_at)	-0.499
HDAC1	-0.617	-0.669 (201209_at)	-0.594
SLC30A7	-0.659	-0.627 (226601_at)	-0.615
FAM46A	-0.612	-0.676 (224973_at)	-0.580
GNG5	-0.643	-0.640 (207157_s_at)	-0.553
NEDD1	-0.610	-0.644 (1560116_a_a)t	-0.659
AK2	-0.652	-0.633 (208967_s_at)	-0.531
MBD2	-0.606	-0.715 (202484_s_at)	ND
CDK2	-0.604	-0.623 (204252_at)	-0.523
PLOD1	-0.607	-0.611 (200827_at)	-0.498
