# Supplementary material for: LncRNA DGCR5 plays a tumor-suppressive role in glioma via the miR-21/Smad7 and miR-23a/PTEN axes
Source: Aging (Albany NY). 2020 Oct 21;12(20):20285–307. doi: 10.18632/aging.103800 (PMC7655220; doi:10.18632/aging.103800)
Supplement: Supplementary Figures [file aging-12-103800-s002..pdf]

SUPPLEMENTARY FIGURES

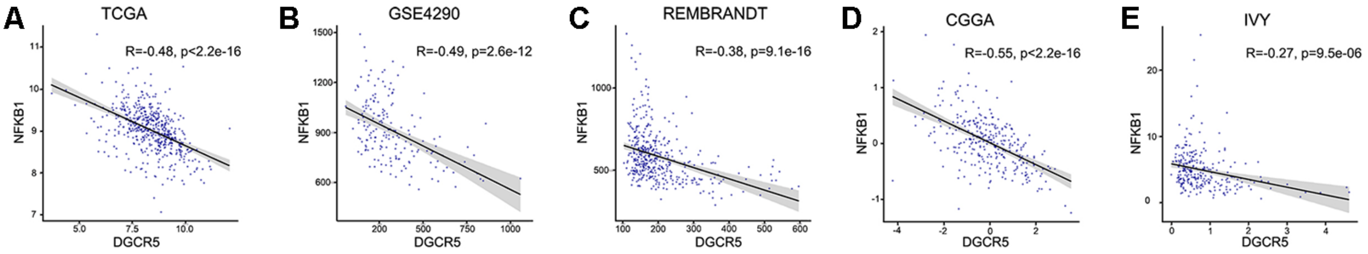

Supplementary Figure 1. Correlation between DGCR5 and NF-κB1 according to (**A**) TCGA data, (**B**) GSE4290, (**C**) REMBRANDT, (**D**) CGGA, and (**E**) IVY.

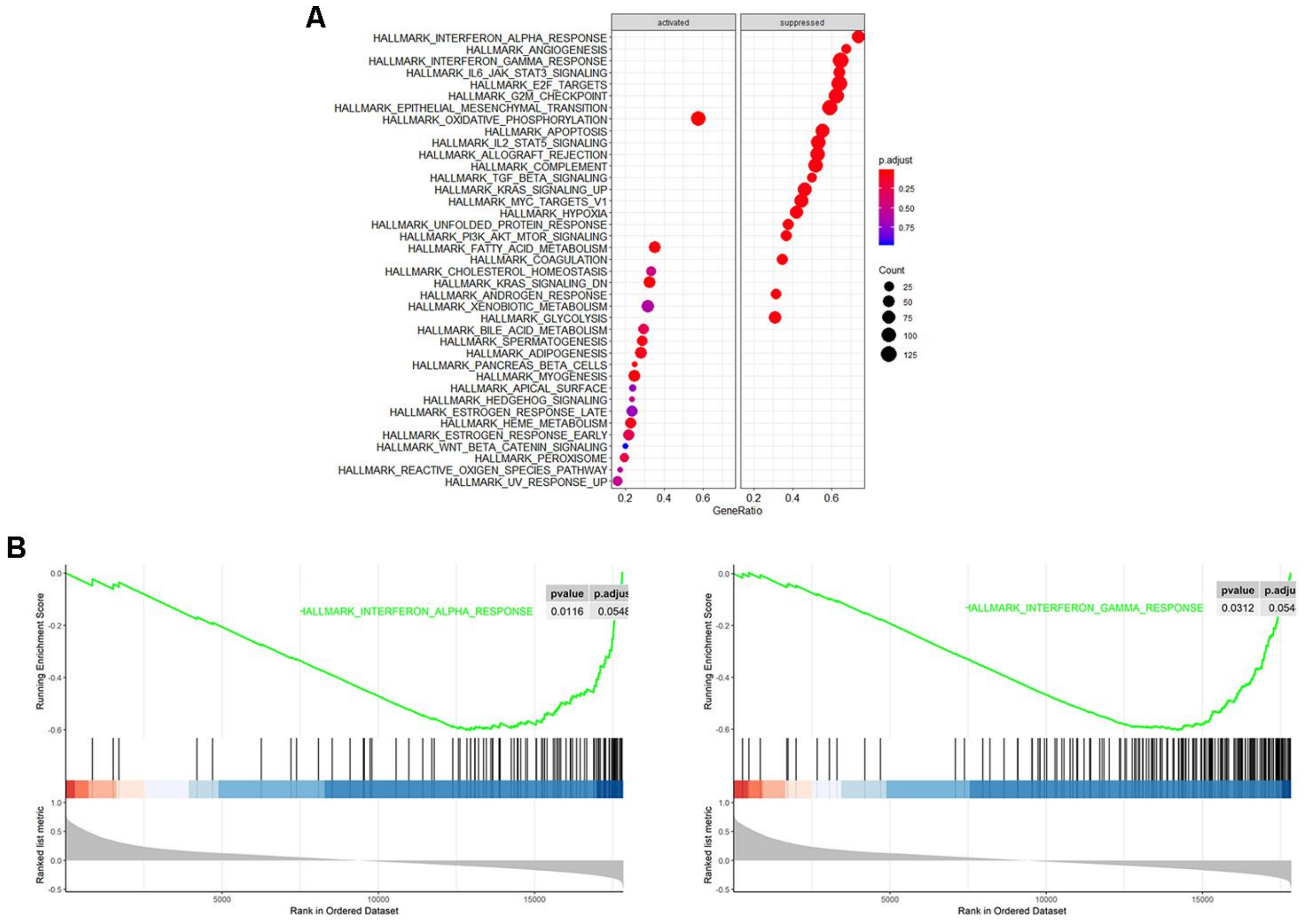

Supplementary Figure 2. Kyoto Encyclopedia of Genes and Genomes (KEGG) (**A**) and Gene Set Enrichment Analysis (GSEA) (**B**) on DGCR5.

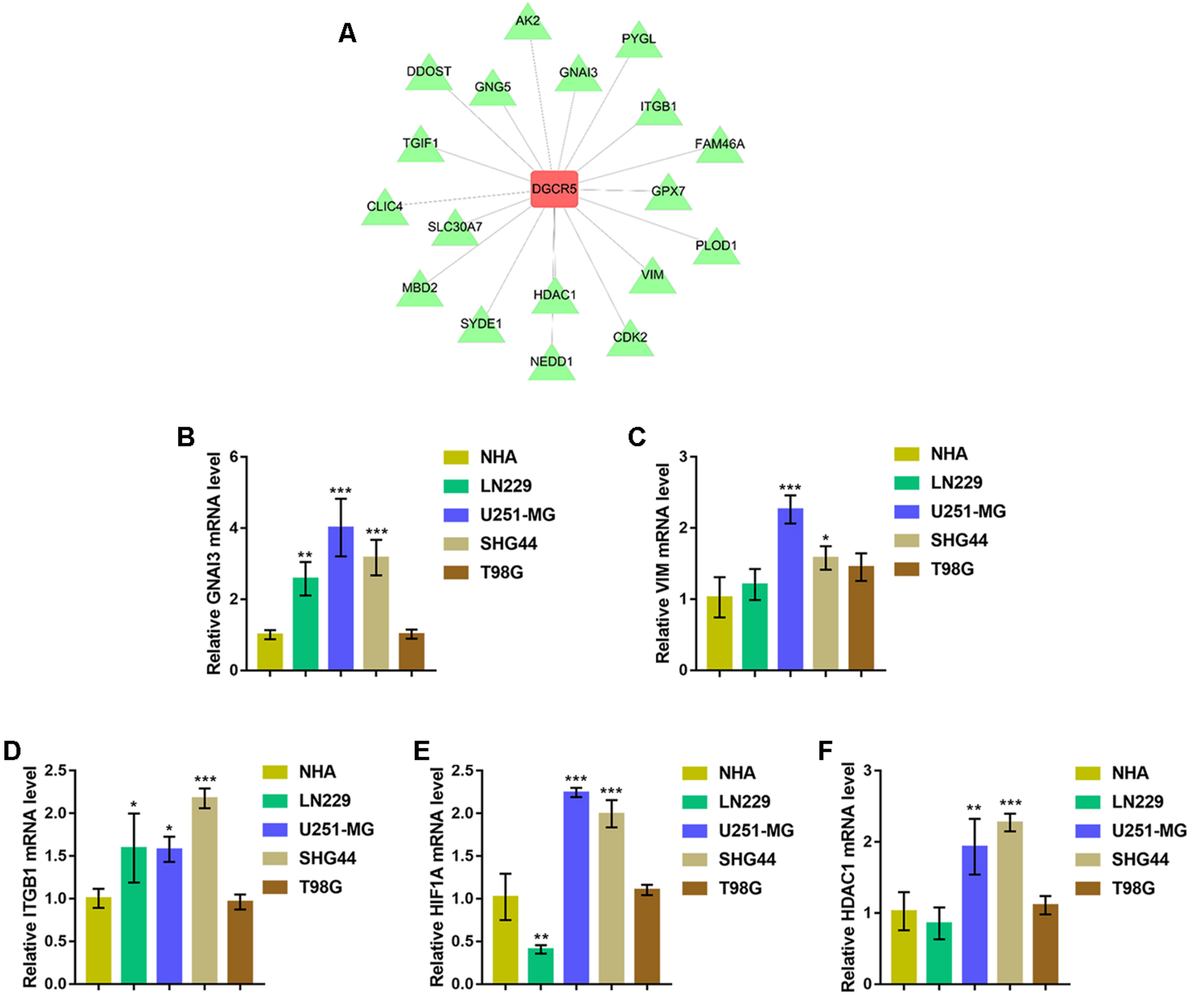

Supplementary Figure 3. Co-expression network consists of DGCR5 and its negatively-correlated genes (**A**) Co-expression network consists of DGCR5 and its negatively-correlated genes. (**B**–**F**) Expression of genes negatively-correlated with DGCR5 in NHA, LN229, U251-MG, SHG44, and T98G cells. **P*<0.05, ***P*<0.01, ****P*<0.005.

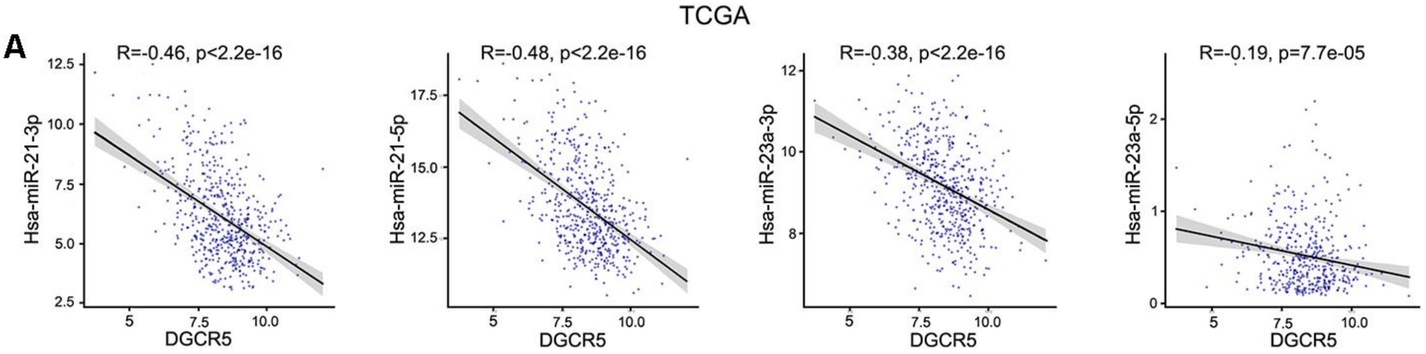

Supplementary Figure 4. Correlation between DGCR5, miR-21, and miR-23a according to TCGA data.

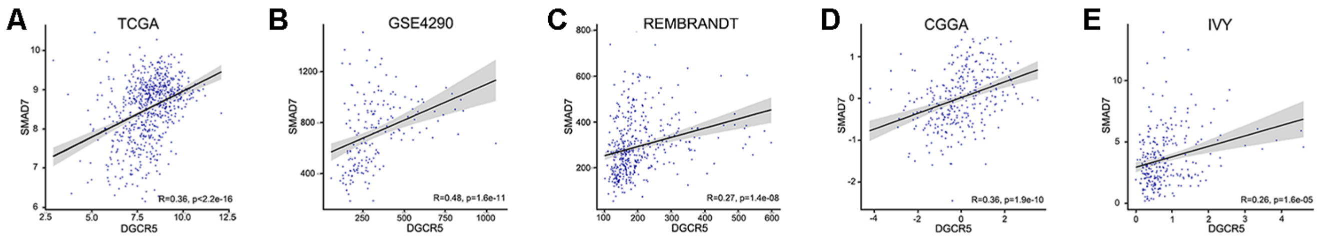

Supplementary Figure 5. Correlation between DGCR5 and Smad7 according to (**A**) TCGA data, (**B**) GSE4290, (**C**) REMBRANDT, (**D**) CGGA, and (**E**) IVY.

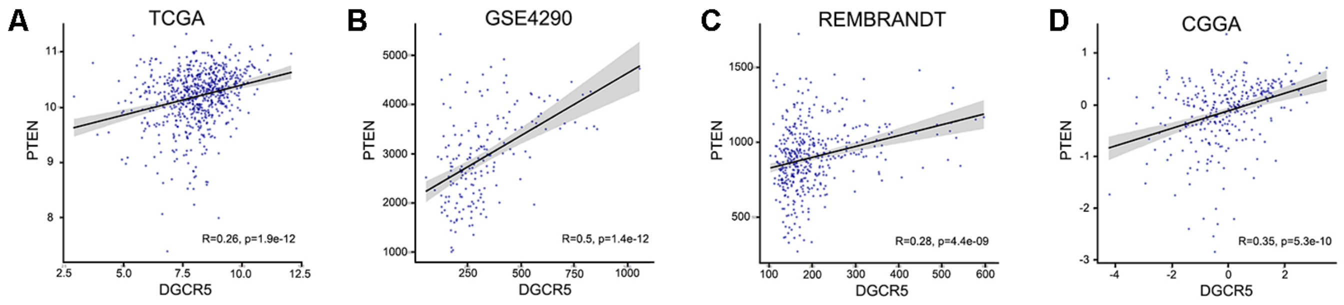

Supplementary Figure 6. Correlation between DGCR5 and PTEN according to (**A**) TCGA data, (**B**) GSE4290, (**C**) REMBRANDT, and (**D**) CGGA.
